# Optimizing the performance of convolutional neural network for enhanced gesture recognition using sEMG

**DOI:** 10.1038/s41598-024-52405-9

**Published:** 2024-01-23

**Authors:** Hassan Ashraf, Asim Waris, Syed Omer Gilani, Uzma Shafiq, Javaid Iqbal, Ernest Nlandu Kamavuako, Yaakoub Berrouche, Olivier Brüls, Mohamed Boutaayamou, Imran Khan Niazi

**Affiliations:** 1 Laboratory of Movement Analysis (LAM-Motion Lab), University of Liège, Liège, Belgium; 2 Department of Biomedical Engineering and Sciences, School of Mechanical and Manufacturing Engineering (SMME), National University of Science and Technology (NUST), Islamabad, 44000 Pakistan; 3 Department of Electrical, Computer and Biomedical Engineering, Faculty of Engineering, Abu Dhabi University, Abu Dhabi, United Arab Emirates; 4 Department of Informatics, King’s College London, London, WC2R 2LS UK; 5 LIS Laboratory, Department of Electronics, Faculty of Technology, Ferhat Abbas University Setif 1, Setif, Algeria; 6 New Zealand College of Chiropractic, Auckland, New Zealand

**Keywords:** Computational models, Data processing

## Abstract

Deep neural networks (DNNs) have demonstrated higher performance results when compared to traditional approaches for implementing robust myoelectric control (MEC) systems. However, the delay induced by optimising a MEC remains a concern for real-time applications. As a result, an optimised DNN architecture based on fine-tuned hyperparameters is required. This study investigates the optimal configuration of convolutional neural network (CNN)-based MEC by proposing an effective data segmentation technique and a generalised set of hyperparameters. Firstly, two segmentation strategies (disjoint and overlap) and various segment and overlap sizes were studied to optimise segmentation parameters. Secondly, to address the challenge of optimising the hyperparameters of a DNN-based MEC system, the problem has been abstracted as an optimisation problem, and Bayesian optimisation has been used to solve it. From 20 healthy people, ten surface electromyography (sEMG) grasping movements abstracted from daily life were chosen as the target gesture set. With an ideal segment size of 200 ms and an overlap size of 80%, the results show that the overlap segmentation technique outperforms the disjoint segmentation technique (p-value < 0.05). In comparison to manual (12.76 ± 4.66), grid (0.10 ± 0.03), and random (0.12 ± 0.05) search hyperparameters optimisation strategies, the proposed optimisation technique resulted in a mean classification error rate (CER) of 0.08 ± 0.03 across all subjects. In addition, a generalised CNN architecture with an optimal set of hyperparameters is proposed. When tested separately on all individuals, the single generalised CNN architecture produced an overall CER of 0.09 ± 0.03. This study's significance lies in its contribution to the field of EMG signal processing by demonstrating the superiority of the overlap segmentation technique, optimizing CNN hyperparameters through Bayesian optimization, and offering practical insights for improving prosthetic control and human–computer interfaces.

## Introduction

The development of improved myoelectric prosthesis control systems is receiving a surge in attention as a result of recent developments in machine learning (ML), deep neural networks (DNNs) and rehabilitation technology. Surface electromyogram signal (sEMG) signals are used to detect hand motion, and this method is regarded as fundamental in the literature^1^. Through the analysis of sEMG data, traditional ML algorithms such as support vector machines (SVMs) and linear discriminant analysis (LDA) have been employed to identify the intended hand^2^. Although conventional pattern-recognition-based myoelectric control has received much scholarly attention over the last few decades, cutting-edge approaches have not been applied in many practical commercial applications^3^. This is due, among many others, to the intrinsic data-driven nature of the ML and DNN algorithms. Regardless of the MEC configuration, the MEC’s success depends greatly on the classification accuracy, which is strongly correlated with the selection of the MEC parameters and hyperparameters of the DNN algorithms. To achieve the best performance results, mainly two types of parameters need to be tuned:

### MEC parameters

The number and location of sEMG channels, sampling frequency, segmentation technique, and segment size length contribute to any MEC system's success. Each of these parameters has been extensively investigated for classical ML-based MEC systems. However, only a few studies have been conducted to study the effect of these parameters on a DNN-based MEC.

### Hyperparameters of the DNN algorithm

Many ML/DL methods rely on hyperparameter selection, and these selections have a significant influence on system performance. The authors in Ref.^4^ thoroughly investigated a single highly parameterised model family, providing classification performance ranging from a chance to state-of-the-art performance, based simply on hyperparameter selection. This and other recent studies demonstrate that the question of "How good is this model on that dataset?" is incorrectly posed. Instead, the quality of the optimal configuration that a specific search technique can typically identify in a given period for a job at hand makes sense. According to this viewpoint, hyperparameter adjustment is a key aspect of understanding algorithm performance and should be a formal and quantifiable part of model assessment.

Hyperparameter optimisation or tuning is the identification of a set of hyperparameter settings, before the training phase, that archive the greatest data performance in a fair amount of time. The choice of hyperparameters is critical for the performance of ML frameworks. Unfortunately, the link between machine learning algorithm performance and hyperparameters is uncertain. Numerous ML models are trained by utilising various combinations of hyperparameters to identify the set of hyperparameters for an optimal ML model.

Hyperparameter optimisation techniques can broadly be categorised as manual, grid, random, and automatic optimisation search. Besides the automatic optimisation search method, all the other optimisation techniques iteratively traverse the entire space of accessible parameter values to identify the best possible combination of hyperparameters. Tuning using these approaches may be time-consuming, especially with large parameter spaces. The search space expands exponentially as the number of tuning parameters increases. At the same time, for each hyperparameter combination, a model must be trained, predictions must be made using validation data, and the validation metric must be determined. Hyperparameter tuning by automated optimisation search can reduce the time required to identify the best set of parameters and improve generalisation performance on the test set.

Bayesian optimisation is an automated optimisation technique for the optimisation of complex functions. In contrast with the other global optimisation algorithms, Bayesian optimisation performs better optimisations for a wide range of complex functions^5^. The classical Bayesian theorem helps to estimate the posterior information on the function distribution by combining the sample information with prior information on the unknown function. This posterior information leads to identifying the optimal values of the function under consideration. We hypothesise that the Bayesian optimisation can not only be used to identify the hyperparameters of the DNN model but also the architecture of the DNN model can be optimised. The identified architecture and hyperparameters can be then used to construct an ideal MEC for sEMG-based hand gesture recognition, which might address the challenges of limited generalisation and a large training burden that have hampered the development of myoelectric control technology.

This study presents the notion of Bayesian optimisation for MEC systems for the first time. The proposed Bayesian optimisation considers the previous evaluations of the objective function before evaluating the next set of hyperparameters. This intelligent approach makes it possible to focus on those areas of the parameter space that may provide the most promising performance results. Bayesian optimisation can ignore the parameter space areas that do not contribute to reducing the objective function. This ability and consideration of previous evaluations make it possible to find the optimal set of hyperparameters with fewer iterations. The following contributions are made to the paper:

1. An extensive experiment has been conducted to investigate which segmentation technique (overlap and disjoint), and segment size provides the best variance-bias trade-off to design a DNN-based MEC.
2. A completely optimised CNN architecture, as well as hyperparameter possibilities, have been discovered using Bayesian optimisation. The proposed optimisation approach not only shortens the time required to discover the optimum DNN hyperparameters but also mitigates the significant challenge of reproducibility of the DNN results.

The structure of the paper is as follows: Section "Related work" provides an overview of the related work. In Section "Principle of Bayesian optimisation", the principle of Bayesian optimization is explained. Section "Methods" outlines the methodology employed in this study. The findings of the study are presented in Section "Results". In Section "Discussion", a detailed discussion of the results is provided, and Section "Conclusion" concludes the study.

## Related work

In recent research, a typical technique for hand gesture identification is to employ DNN to increase hand gesture classification performance on "unseen data." Several cutting-edge efforts in this field mostly employed 1D and 2D DNNs^4,6^. Pre-processing, segmentation, feature extraction, and classification are typical pipeline steps for processing sEMG with standard MEC systems. DNNs, on the other hand, often use raw sEMG signals as input and multi-layer nonlinear functions to represent the hidden connections between the input and the output. It is important to note that the performance of ML and DNN is affected by various aspects, including pre-processing and the specified architecture. Other academic researchers, as well as our team, have recently conducted several studies aimed at improving the performance of MECs. For example, it has been demonstrated that the performance of an ML-based MEC system is positively correlated with the length of the segment size^7–10^. More precisely, the results reveal that ML-based MEC systems perform best with a segment size of 500 ms. However, regarding a DNN-based MEC, no such investigations have been done. Different studies have utilised different segmentation techniques (either disjoint or overlap) with different segments and overlap sizes. For example, Asif et al. (2020) utilised an overlap segmentation technique with a segment size of 150 ms and an overlap size of 25 ms to segment the sEMG signals from data recorded with a sampling frequency of 8000 Hz^4^. Another study reported using an overlap segmentation technique with a segment size of 150 ms and an overlap of 100 ms to design an adaptive domain adversarial neural network to recognise sEMG hand gestures^11^. Similarly, Rahimian et al. (2021) proposed a few-shot learning-based DNN framework for the MEC system and utilised a disjoint segmentation technique with a segment size of 50 ms^12^. Chen et al. (2020) proposed a transfer learning-based MEC system and processed the sEMG signals with an overlap segmentation technique having a segment size of 100 ms and an overlap size of 50 ms^13^. In another study, the authors utilised a disjoint segmentation technique with a segment size of 260 ms to recognise sEMG hand gestures using a compact CNN^14^.

Consequently, there have been few efforts to design advanced DNN architectures for MEC with optimised hyperparameters. In Ref.^4^, the impact of the learning rate and the number of epochs for a CNN-based MEC. The results showed that, for a MEC, the CNN performs best with a learning rate set to either 0.0001 or 0.001 with 80–100 epochs. Another study investigated the effect of the number of sEMG channels, the number of filters, and the choice of the optimiser on the performance of CNN-based MEC^15^. It was shown that the CNN architecture with Adam optimiser, having the number of convolutional filters in the range from 100 to 200, and the maximum number of sEMG channels resulted in the highest classification accuracy. It is worth noting that both studies conducted an empirical investigation to identify optimal parameters. Only one study has reported the use of an optimisation algorithm (crow search metaheuristic algorithm) for the identification of DNN hyperparameters^16^. However, the study was conducted on the hand gesture recognition database composed of a set of near-infrared images.

It is worth noting that while it is true that various previous studies have achieved excellent performance in sEMG gesture classification without extensive MEC and hyperparameter tuning, our study places a deliberate emphasis on hyperparameter optimization for several reasons: (1) Different applications and datasets may have unique characteristics, noise levels, and requirements. MEC and hyperparameter optimization allows us to fine-tune the CNN model to best fit the specific context of the dataset, potentially yielding improved classification accuracy in scenarios where such fine-tuning is critical. (2) By exploring hyperparameter optimization, our study provides valuable insights into how to improve model generalizability. This information can be particularly useful for researchers and practitioners seeking to apply CNN-based EMG signal processing techniques to a variety of real-world applications. (3) Our research serves as a benchmark for evaluating the potential impact of hyperparameter optimization in the context of HD-sEMG gesture classification. It allows researchers to compare the performance of models with and without hyperparameter tuning, thereby helping to establish best practices and guidelines in the field. In summary, while some prior works have indeed achieved strong results without extensive MEC and hyperparameter tuning, our study intentionally focuses on this aspect to explore its impact on classification accuracy and to provide insights that can be applied to a range of EMG signal processing applications. We believe this emphasis on hyperparameter optimization enriches the existing body of knowledge in the field and complements the achievements of prior studies.

The research gap in the context of MEC research primarily lies in the limited exploration of key factors that influence the data preprocessing and hyperparameter optimization for CNNs. While previous studies have mainly concentrated on DNN architectures for hand gesture recognition, there has been a paucity of comprehensive investigations into the segmentation techniques (overlap and disjoint) and the segment size, which play a pivotal role in the data preprocessing pipeline. This research contributes by addressing this notable gap in the field, delving deeply into the effects of segmentation techniques and segment size on DNN-based MEC, thus unveiling a novel dimension in data preprocessing. Moreover, the study introduces a groundbreaking approach to hyperparameter optimization through Bayesian optimization, which offers a streamlined and efficient method for discovering the most effective CNN architectures and hyperparameter settings. The integration of Bayesian optimization addresses the challenge of reproducibility in DNN results and establishes a new paradigm in optimizing DNNs for MEC systems, making it a pivotal and innovative contribution to the existing body of knowledge.

## Principle of Bayesian optimisation

### Problem formulation

Hyperparameter optimisation aims to identify the optimal set of hyperparameters for a specific ML/DNN model which can provide the best performance results upon the evaluation. Mathematically, hyperparameter optimisation is expressed as ${x}^{*}=\genfrac{}{}{0pt}{}{argmin}{x \in \chi }f(x)$. Here $x$ can take on any value in the domain $\chi$; ${x}^{*}$ is the set of identified hyperparameters providing the lowest classification error, and $f(x)$ is the classification error (objective function) which needs to be minimized.

### Overview of Bayesian optimization

**Figure 1:**
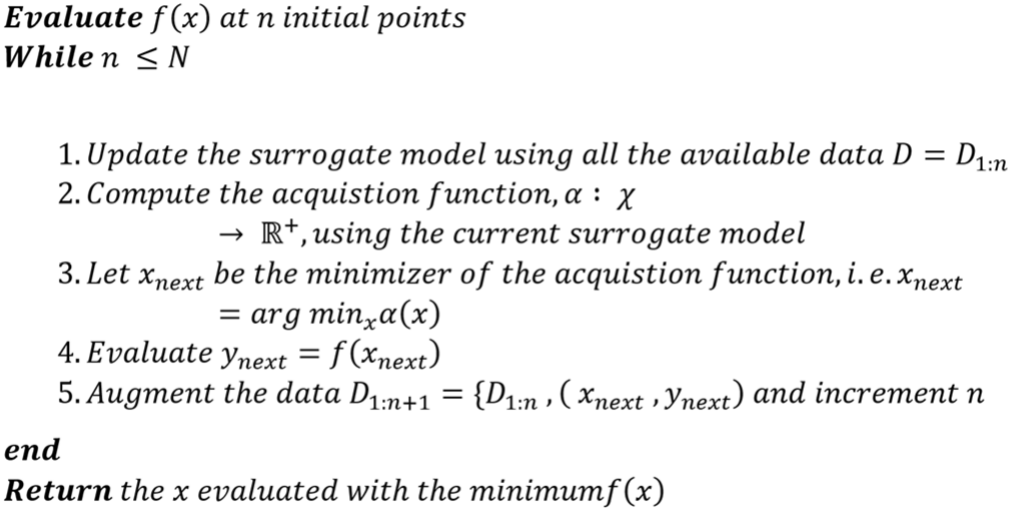
Pseudocode for the Bayesian optimization. Here $f(x)$ is the objective function from a subset of ${\mathbb{R}}^{D}$ on some bounded set $\chi$. Where D is the set of available N data points. Whereas $\alpha (x)$ is the acquisition function.

The Bayesian optimisation aims to minimise $f(x)$ from a subset of ${\mathbb{R}}^{D}$ on some bounded set $\chi$. Firstly, a probabilistic model for $f(x)$ is built to estimate the subsequent evaluations from $\chi$ while eliminating the uncertainties. Bayesian optimisation takes advantage of local gradient and Hessian approximations and also utilises the information from prior evaluations of the objective function^17^. This results in the identification of the minima of complex non-convex optimisation problems; however, additional calculations are needed to estimate the location of the next evaluation in the subspace. The results of previous evaluations are used to build a surrogate function denoted by $p(x|y)$. The surrogate function is a probabilistic model mapping hyperparameters to the likelihood of a score on the objective function. Minimising the surrogate function is comparatively more straightforward than the objective function^18^. The hyperparameters that perform best on the surrogate function are then used to determine the next set of hyperparameters to minimise the true objective function under consideration. Figure 1 presents the pseudocode for Bayesian optimisation. Here are the key components:

1. *Search space*: Bayesian optimisation works by sampling from probability distributions for each parameter. The user must configure these distributions. One of the subjective aspects of the procedure is determining the distribution for each parameter. The initial learning rate, network depth, stochastic gradient descent momentum, and L2 regularisation strength have been considered as optimising parameters.
2. *Objective function*: The objective function is the primary assessor of hyperparameter combinations. It simply accepts a set of hyperparameters and returns a score indicating how well the collection of hyperparameters performs on the validation set. The classification error will be our preferred assessment statistic for our hand gesture classification challenge. The goal in this scenario is to minimise the objective function. The fundamental idea behind Bayesian optimisation is to limit the number of times the objective function must be run by evaluating just the most promising set of hyperparameters based on past calls to the evaluation function. The next set of hyperparameters is chosen on a surrogate model.
3. * Surrogate function*: The surrogate function is the probability representation of the objective function constructed from past assessments. It is a high-dimensional representation of hyperparameters to the probability of scoring well on the objective function. The objective function can be approximated using the surrogate function. It is used to suggest parameter choices to the objective function that are likely to increase the accuracy score. The Gaussian process (GP) is the most commonly used surrogate function due to its flexibility and tractability^18,19^.
4. * Acquisition function*: The acquisition or selection function is the set of conditions used to determine the subsequent combination of surrogate function hyperparameters. The hyperparameters that are put forth for assessment by the objective function in Bayesian optimisation are chosen by applying criteria to the surrogate function. A selection function defines this criterion. A popular strategy is to employ a metric known as expected improvement (EI)^20^.

### Bayesian optimisation

Suppose that the objective function $f(x)$, is chosen from a GP and that the observations are of the form ${\{{x}_{n} , {y}_{n}\}}_{n=1}^{N}$, with predictive mean function $\mu (x;\left\{{x}_{n} ,{y}_{n}\right\},\theta )$ and predictive variance function ${\sigma }^{2}(x;\left\{{x}_{n} ,{y}_{n}\right\},\theta )$^17^. Here, $\theta$ indicates the vector of the kernel functions. This prior and these data provide a posterior over functions denoted by $\alpha :\chi \to {\mathbb{R}}^{+}$ and known as the acquisition function. The acquisition function determines, with the help of proxy optimisation ${x}_{next}={argmin}_{x}\alpha (x)$, which point in $\chi$ should be evaluated next in which multiple functions have been offered^18^. The GP hyperparameters and results of the past evaluations drive the acquisition function. Mathematically this relationship is referred as $\alpha (x;\left\{{x}_{n} ,{y}_{n}\right\},\theta )$. The expected improvement in the optimisation of the $f(x)$ is monitored by EI while ignoring the values from the subspace which are not contributing to the optimisation. The EI under GP may be summarised as follows:

1 $$EI \left(x,Q\right)= {E}_{Q}[{\text{max}}(0,{\mu }_{Q}\left({x}_{best}\right) -f(x))].$$

Here, ${\mu }_{Q}\left({x}_{best}\right)$ represents the lowest value of the posterior mean. The time it takes to optimise the objective function might vary depending on the search space^19^. If this is the case, integrating time-weighting into the acquisition function will result in a better improvement per second. To do this, another Bayesian model of $f(x)$ as a function of location $x$ is maintained throughout the objective function evaluations. The anticipated improvement per second (EIpS) may be calculated:

2 $$EIpS\left(x\right)= \frac{{EI}_{Q}(x)}{{\mu }_{S}(x)}.$$

Here ${\mu }_{S}(x)$ represents the posterior mean of the GP model. The acquisition function predicts the overexploitation of an area to avoid the minima of local $f(x)$. If $\sigma$ denotes the posterior standard deviation of the additive noise and ${\sigma }_{F}(x)$ is the standard deviation of the posterior $f\left(x\right)$ at $x$, then according to ${\sigma }_{Q}^{2}\left(x\right)= {\sigma }_{F}^{2}\left(x\right)+{\sigma }^{2}$ local minima can be avoided^19^. To do so, the acquisition function evaluates if the next point $x$ satisfies ${\sigma }_{F}\left(x\right)< \sigma {t}_{\sigma }$, after each iteration. Here ${t}_{\sigma }$ denotes a positive number representing the value of the exploration ratio. The exploration ratio governs the trade-off between investigating new locations for a better overall solution and focusing on previously investigated places. If the requirement is met, the algorithm determines $x$ to be overexploited. The acquisition function then adjusts its kernel function by multiplying $\theta$ by the iteration count. This change increases the variation for points between observations. The newly fitted kernel function is then used to construct a new point.

**Figure 2:**
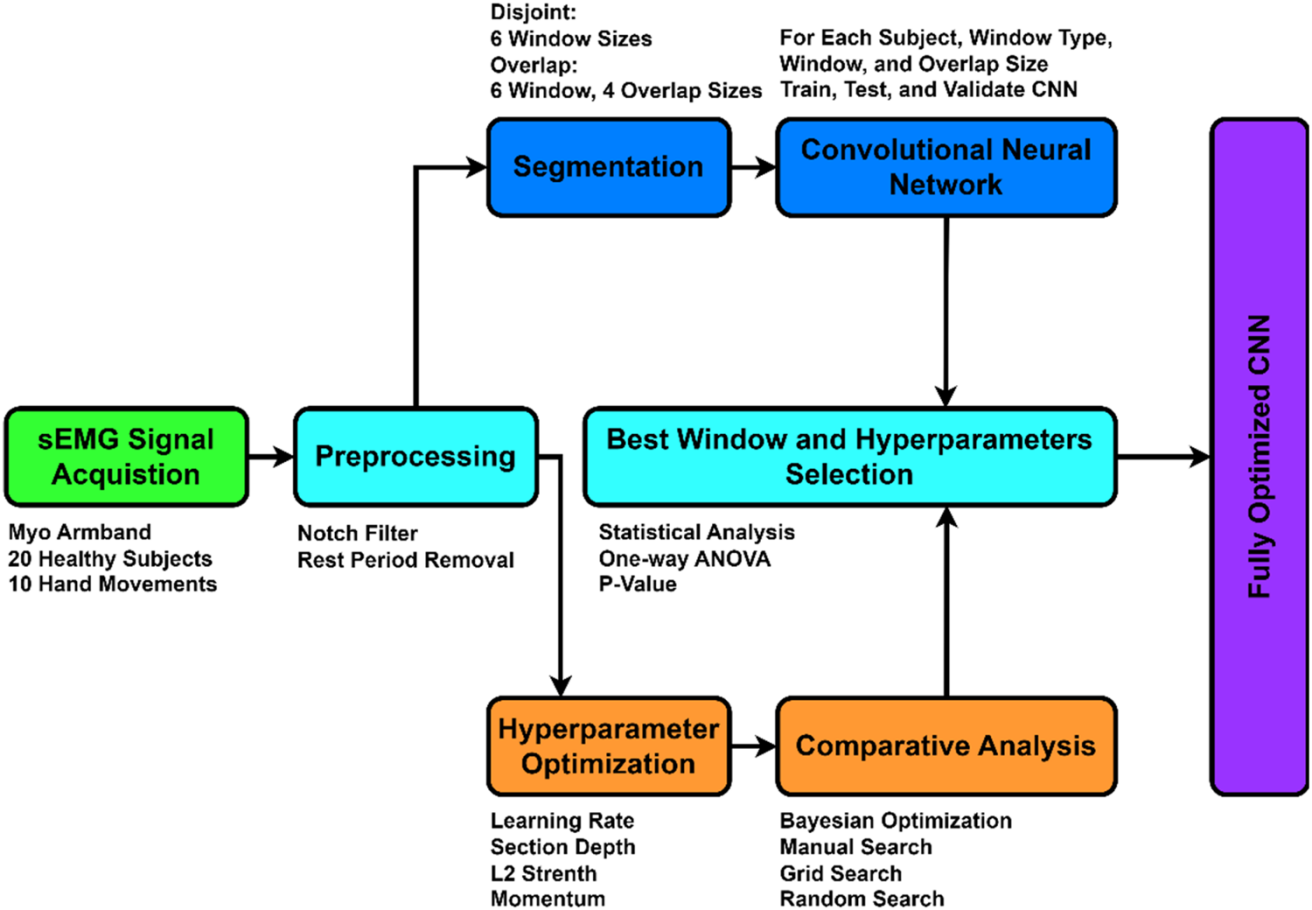
Block diagram depicting different steps of the study.

The overall procedure of the study is depicted in Fig. 2.

## Methods

### Dataset

Informed consent from all the subjects was obtained before the start of the experiment. All the subjects were adults and provided their written consent. The Myo dataset, a new, publicly accessible (available at: https://shorturl.at/ghqLZ) sEMG-based hand gesture recognition dataset, is one of the article's significant contributions. The dataset, which includes 20 healthy volunteers (all males; average age (years) = 28.6 ± 1.4; average height (cm) = 168.0 ± 7.8; average weight (kg) = 68.9 ± 8.6), should be used to develop, evaluate, and optimise sEMG hand gesture classification algorithms. No musculoskeletal disease or prior history of upper extremity disorder was the inclusion criteria for the participants. The dataset was collected using a commercially available Myo Armband from Thalmic Labs, which was previously available for purchase. The dataset includes a diverse set of sEMG signals corresponding to hand gestures performed by the participants. It is organized in a structured format that facilitates the development, evaluation, and optimization of sEMG hand gesture classification algorithms. Researchers can access and utilize this dataset for their own investigations, even if the Myo Armband is no longer available as a new device. We are committed to ensuring the transparency and replicability of our research, and this publicly available dataset is a fundamental part of that commitment. The National University of Sciences and Technology (NUST) ethics committee, Islamabad, Pakistan, authorised the data collecting procedure (approbation number: NUST/SMM-BME/REC/000471/403412022).

The experimental protocol was explained both verbally and in writing, before the experiments, to all the participants. The Myo is an 8-channel, low-cost consumer-grade sEMG armband with a low-sampling rate (200 Hz), dry-electrode, and was used to capture the electromyographic activity from the forearm of each subject. Since the dry electrodes allow users to slip the bracelet on without any preparation, the Myo is non-intrusive. In comparison, gel-based electrodes require shaving and cleaning of the skin to obtain good contact between the electrodes and the participant's skin^20^. Participants wore Myo on their right forearm, covering the muscles of the extensor carpi ulnaris, the flexor carpi radialis, the extensor digitorum, the palmaris longus, the extensor carpi radialis, and flexor digitorum superficialis muscles. Each participant completed a randomised pattern of 10 static and dynamic hand movements during the tests in a single session. Before data collection, each movement was displayed to volunteers using a BioPatRec graphical user interface^21^. In the experiment, the following hand motions were recorded: pointer, agree, fine grip, side grip, supination, pronation, extend hand, flex hand, close hand, and open hand. Each exercise (with 10 repetitions) lasted 10 s, with a contraction duration of 6 s and a relaxation duration of 4 s. For each participant, the data acquisition lasted for 4000 s ([6 s contraction + 4 s rest] × 10 motions × 10 repetitions).

### Pre-processing of data

The acquired sEMG signals were filtered using a second-ordered digital Butterworth notch filter with a cut-off frequency of 50 Hz to minimise the effect of powerline interference. The relaxation or rest period was eliminated from each movement, and only the contraction period was utilised for further processing. Since sEMG signals acquired from the muscles are a continuous stream, these signals are segmented into shorter segments of finite length. Overlap and disjoint segmentation or windowing techniques are primarily employed to segment the sEMG signals. Disjoint segmentation is characterised by segment size, whereas the length of segment size and overlap size characterise overlap segmentation. In a natural daily-life environment, the duration of different hand options differs. For example, dynamic activities' duration is greater than transitional activities' duration. Thus, the length of the segment size should capture enough information to decode the underlying patterns of the intended hand motions. Previously for ML-based MEC systems, it has been shown that overlap segmentation performs better than disjoint segmentation (irrespective of the sampling frequency) also, the performance of the MEC increases with the increasing length of the segment and overlap size^7–9^. However, the real-time application of MEC restricts keeping the length of segment size below 300 ms for the smooth operation of the designed MEC^22^.

For DNN-based MEC systems, however, the effect of segmentation parameters (segmentation type, segment size, and overlap size) on the MEC's performance is unclear. Various researchers have employed different techniques (either overlap or disjoint), segment, and overlap size for DNN-based MEC systems. Thus, it is necessary to identify the effect of segmentation parameters on the performance of a DNN-based MEC. In this study, we have employed both segmentation techniques with different windows (100, 150, 200, 250, 300, and 350 ms) and overlap sizes (20, 40, 60, and 80%) to investigate the effect of segmentation parameters. segmentation parameters.

### Classification of data

A 2D convolutional neural network has been used for the classification of hand motions. Despite the availability of more complicated contemporary CNNs, the choice of a basic CNN was made to speed up the training phase and allow for the evaluation of the impacts of various pre-processing, architectural, and optimisation factors based on the peculiarities of the challenge. CNN creates many feature detectors by itself, called convolutional layers, and sorts the key characteristics necessary to enhance accuracy during training. This is accomplished by converging the filters with input patches, resulting in a receptive field. The classifier requires "spatial and temporal variation" for the network to recognise the input more successfully^23^. The network gains this capability through pooling. It not only helps to minimise distortions but also decreases the dimensionality of the input data, reducing the number of parameters to account for.

Furthermore, pooling is beneficial for extracting dominating characteristics that are rotational and positional invariant, allowing the model to be efficiently trained. As activation functions, rectified linear units (ReLU) were utilised to alleviate the problem of vanishing gradient and enhance training time. Individual filters can use receptive fields to learn with the same weights for all input patches. This field is then passed to the activation function. Finally, a fully connected layer transforms the 2D layer of features into a column vector. This flattened output is fed to a feed-forward neural network, and backpropagation is applied to every iteration of training. Over a series of epochs, the model can distinguish between dominating and certain low-level features in images and classify them using the SoftMax activation function.

**Figure 3:**
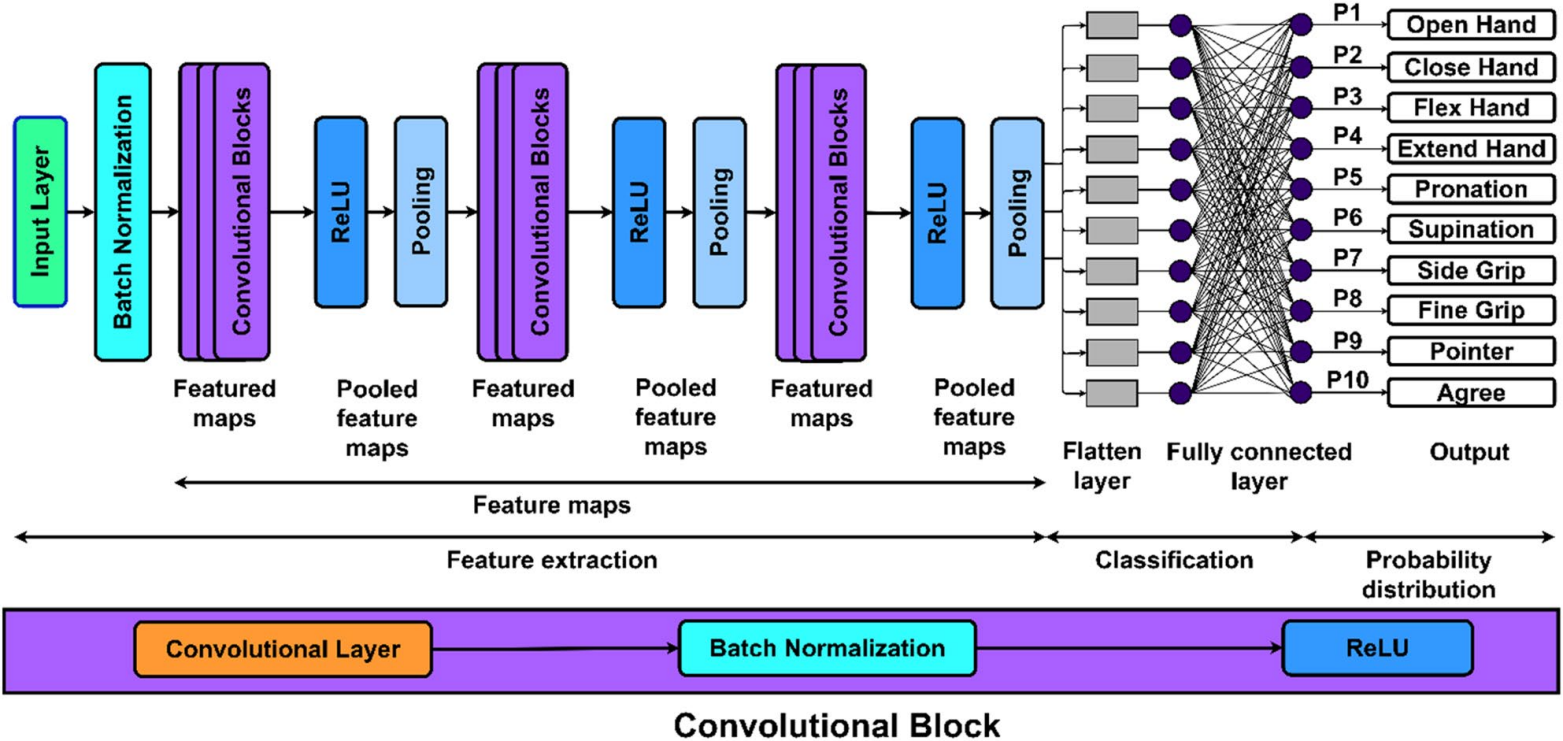
Base architecture of the CNN. In the base architecture, 3 convolutional blocks have been used, where, each convolutional block is comprised of a 2D convolutional, batch normalization, and ReLU layer.

Since CNN uses two-dimensional data arrays as input, the segmented data was transformed into sEMG images. The segmented signals were arranged to create sEMG images of the following dimensions: number of channels × number of samples in a segment. The CNN architecture was designed as shown in Fig. 3.

### Hyperparameters

CNNs yield extraordinary outcomes; training a CNN necessitates mostly empirical approaches for tuning hyperparameters. Because the nature of the signal at hand is entirely random and varies substantially from subject to subject, a generalised set of parameters cannot be derived. However, the selection of these hyperparameters has a significant impact on the outcomes achieved. For complex problems, finding a single learning rate for convergence is hard. Adaptive learning-rate methods are preferred^24^. Despite the availability of recent adaptive optimisers, stochastic gradient descent with momentum (SGDM) has been chosen as an optimiser for CNN to test the capabilities of Bayesian optimisation.

Bayesian optimisation was used to optimise section depth, initial learning rate, stochastic gradient descent momentum, and L2 regularisation strength. The "section depth" option governs the network's depth. The network has three sections, each with identical section-depth convolutional layers. As a result, the total number of convolutional layers is three 3*(section depth). Thus, the number of parameters and quantity of computation required for each iteration is almost the same for varied section depths. Table 1 shows the hyperparameter search space that was chosen.

**Table 1:** The hyperparameter search space for the proposed Bayesian optimization.

Parameter	Search space
Learning rate	[1e − 4 1e − 2]
Stochastic gradient descent momentum	[0.8 0.98]
L2 regularization strength	[1e − 10 1e − 1]
Network section depth	[1 3]

## Results

While the study aimed to optimise both segmentation parameters and CNN hyperparameters, an experiment was initially undertaken to investigate the impact of segmentation parameters on MEC performance. The same architecture as shown in Fig. 1 was used to train, validate, and test the CNN with network section depth (3), learning rate (0.003), momentum (0.8383), L2 regularisation strength (0.0939), and minibatch size (256). All these parameters were empirically determined for all 20 patients. The data for each individual were randomly divided into three categories: training (70%), validation (20%), and testing (10%). To avoid overfitting, the training sEMG images were randomly flipped along the vertical axis and randomly translated up to four pixels horizontally and vertically. The classification error rate (CER) has been obtained for each subject and is defined as the measure of a model's prediction error in comparison to the real labels. CER is represented as a percentage (%). To validate the results, a one-way analysis of variance (ANOVA) test and Tuckey's honest post hoc test were used to determine the statistical significance of the results. A p-value of 5% was considered significant to reject the null hypothesis.

### Segmentation parameters

**Figure 4:**
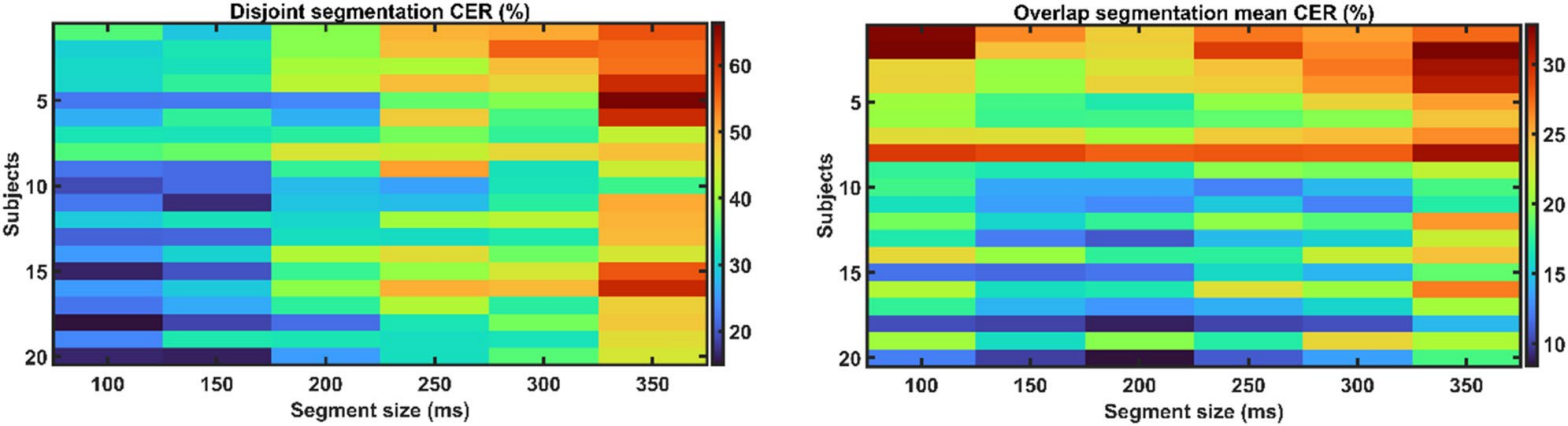
CER (%) vs. segment size for both disjoint and overlap segmentation techniques across all subjects.

Both segmentation strategies with varied segment and overlap sizes were tried for all individuals to determine which segmentation methodology (disjoint or overlap), segment size, and overlap size is most suited to translate sEMG signals into sEMG images considering the performance of the MEC system. Figure 4 shows the CER for all participants using disjoint and overlap segmentation strategies. The CER across four overlap sizes was averaged for overlap segmentation to get the mean CER.

Figure 4 shows that the performance of CNN fluctuates differently depending on the increment in segment size for both segmentation approaches. As the segment length increases for disjoint segmentation, the performance of the CNN gradually decreases. As shown in Fig. 3, the segment sizes of 100 and 150 ms resulted in the lowest CER for all subjects. Based on statistical analysis, it was determined that segment sizes of 100 ms and 150 ms yielded the most significant results, with the lowest mean CER of 25.2% and 26.9%, respectively, and outperformed the other tested segment sizes (p-value < 0.05). However, no significant change in CER between segment sizes of 100 and 150 ms was identified (p-value > 0.05). In contrast, during overlap segmentation, the mean CER drops until the segment size of 200 ms and then begins to increase, as seen in Fig. 4. The segment size of 200 ms resulted in the lowest mean CER of 17.2%. However, there was no significant difference in the mean CER of segment sizes 200 ms and others (p-value > 0.05), except for 350 ms (p-value < 0.05).

**Figure 5:**
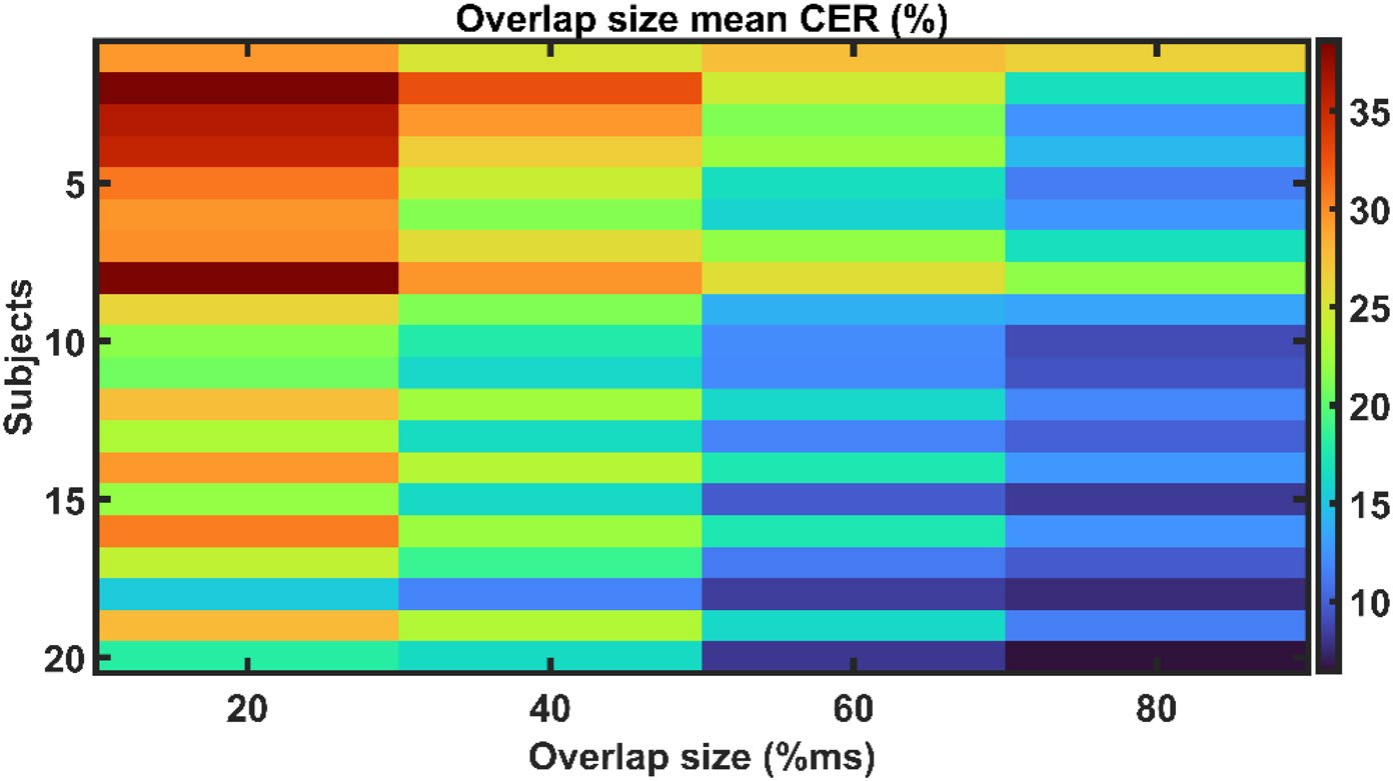
The effect of overlap size on the performance of MEC in terms of CER (%).

**Figure 6:**
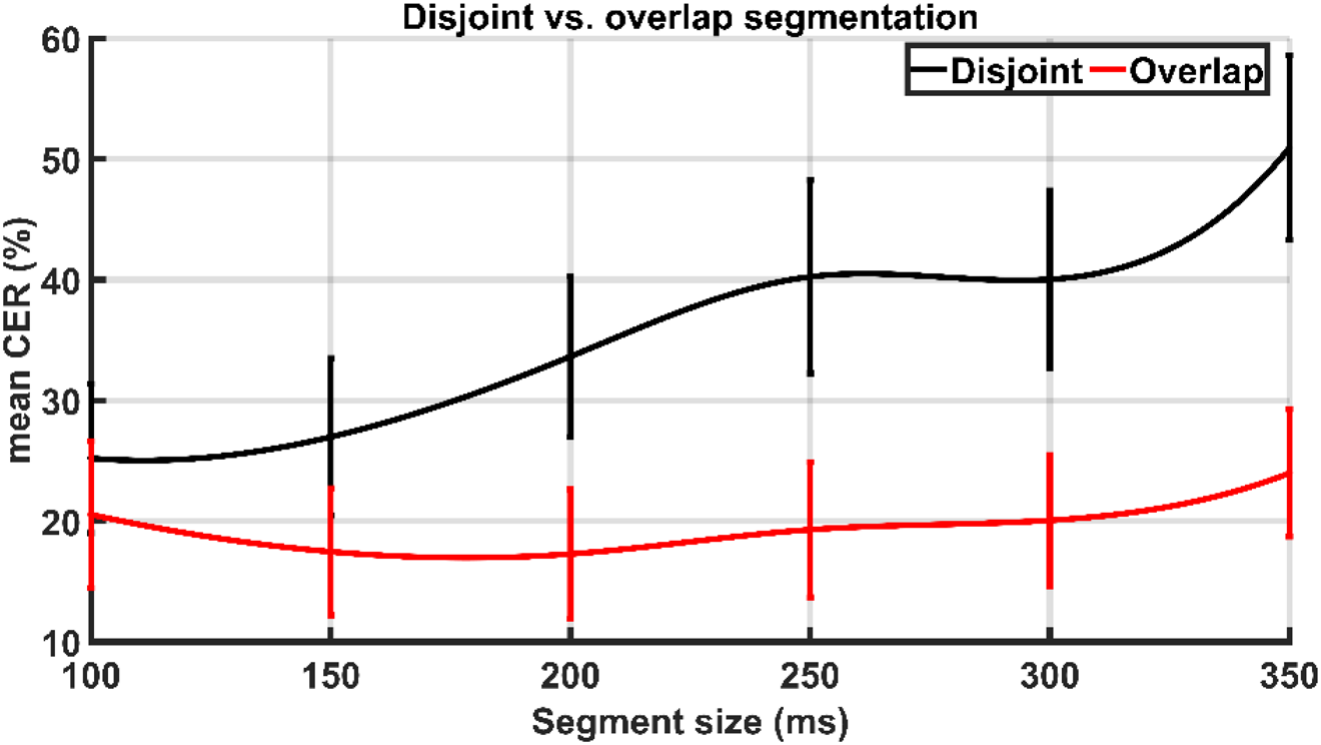
The performance comparison of disjoint and overlap segmentation techniques in terms of mean CER (%). For the overlap segmentation technique, the CER of all overlap sizes has been averaged to get the mean CER.

The influence of overlap size on CNN performance for the overlap segmentation technique has also been explored, and the findings are shown in Fig. 5. The performance of the CNN improves with increasing overlap size across all subjects. The overlap size of 80% resulted in the lowest mean CER of 12.7%, outperforming the overlap sizes of 20% and 40% (p-value < 0.05). There was no significant difference in performance between overlap sizes of 60% (mean CER: 16.5%) and 80% (mean CER: 12.7%) (p-value > 0.05). The mean CER of disjoint and overlap segmentation approaches is shown in Fig. 6. Observing the results, it is evident that the mean CER shows an upward trend as the segment size increases in disjoint segmentation. In contrast, during overlap segmentation, the mean CER declines until the segment size of 200 ms, at which point it begins to increase. The overlap segmentation outperforms the disjoint segmentation statistically (p-value < 0.05).

### Hyperparameter optimization

The optimal segmentation parameters obtained from the trial findings (segmentation technique = overlap; segment size = 200 ms; overlap size = 80%) were used in a subsequent Bayesian optimisation experiment. In Bayesian optimisation, identifying the hyperparameter search space is a subjective job that substantially impacts the results. The number of function evaluations determines the amount of time required to optimise the CNN using Bayesian optimisation. Thus, it is critical to integrate domain knowledge to achieve better optimisation results with fewer function evaluations. The hyperparameter search space selected is based on expert subject knowledge^4–7^. The number of function evaluations in this experiment was set at 30.

Table 2 displays the results of the Bayesian optimisation for all the subjects. A mean CER of 0.08 ± 0.03 was obtained on the testing set. Furthermore, a comparison of the CERs for validation, testing, and 95% confidence interval testing demonstrates that the improved CNN fits the data without over or underfitting.

**Table 2:** Bayesian optimization results for all subjects. The results are presented in terms of classification error rate (%).

Subject	Validation	Testing	95CI testing	
1	0.08	0.10	0.08	0.11
2	0.10	0.11	0.09	0.13
3	0.09	0.12	0.10	0.14
4	0.10	0.12	0.10	0.13
5	0.06	0.07	0.05	0.08
6	0.08	0.09	0.07	0.11
7	0.11	0.12	0.10	0.14
8	0.15	0.18	0.16	0.21
9	0.08	0.08	0.07	0.10
10	0.06	0.06	0.05	0.08
11	0.06	0.06	0.04	0.07
12	0.08	0.09	0.07	0.11
13	0.07	0.07	0.05	0.08
14	0.08	0.09	0.07	0.10
15	0.03	0.04	0.03	0.05
16	0.07	0.08	0.07	0.10
17	0.05	0.06	0.04	0.07
18	0.03	0.05	0.04	0.06
19	0.06	0.08	0.06	0.09
20	0.03	0.05	0.03	0.06
Mean ± std	0.07 ± 0.03	0.08 ± 0.03	0.07 ± 0.03	0.10 ± 0.04

### Optimal hyperparameters

Table 3 shows each subject's optimal hyperparameter combinations determined through Bayesian optimisation. The supplied viable hyperparameters are acquired solely from the validation set, and the same hyperparameters are then utilised to assess the model's performance using the testing set. The results in Table 2 show that optimised hyperparameters reduce generalisation error quite well. Over iterations, the optimisation was carried out in the graphical processing unit (GPU). Every layer optimises to minimise the objective function.

**Table 3:** The identified sets of hyperparameters for all the subjects resulted from Bayesian optimization.

Subject	Learning rate	Momentum	L2 strength	Section depth
1	0.0007	0.9795	4.06E − 02	2
2	0.0098	0.8897	1.59E − 07	3
3	0.0014	0.8948	7.36E − 02	3
4	0.0026	0.9703	1.32E − 05	2
5	0.0069	0.8826	2.94E − 10	2
6	0.0100	0.8028	4.85E − 05	2
7	0.0027	0.9762	1.32E − 10	2
8	0.0094	0.8580	3.91E − 09	3
9	0.0024	0.9697	6.05E − 04	2
10	0.0097	0.8041	1.07E − 10	2
11	0.0018	0.9656	8.59E − 03	2
12	0.0063	0.9160	2.21E − 08	3
13	0.0056	0.8660	2.98E − 10	3
14	0.0017	0.9795	1.45E − 10	2
15	0.0030	0.9365	7.16E − 07	2
16	0.0021	0.9799	1.42E − 06	2
17	0.0090	0.8036	1.10E − 05	3
18	0.0014	0.9781	1.14E − 10	3
19	0.0050	0.8591	2.80E − 03	2
20	0.0056	0.8039	6.78E − 02	2

**Figure 7:**
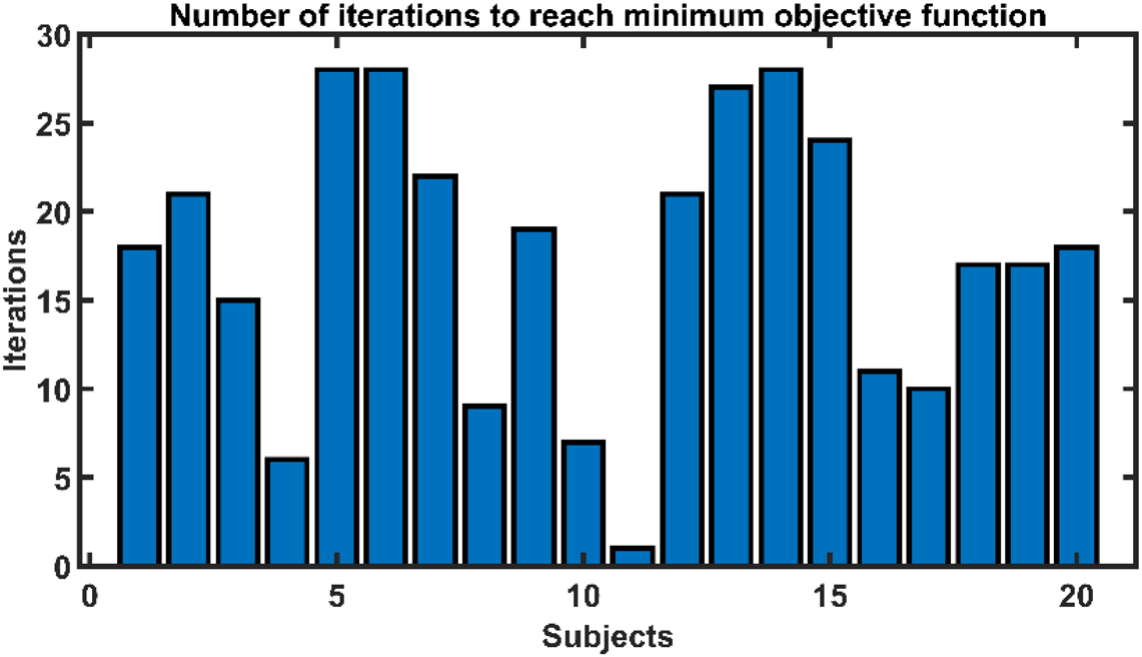
The number of iterations to reach the minimum objective function during hyperparameter optimization using Bayesian optimization.

**Figure 8:**
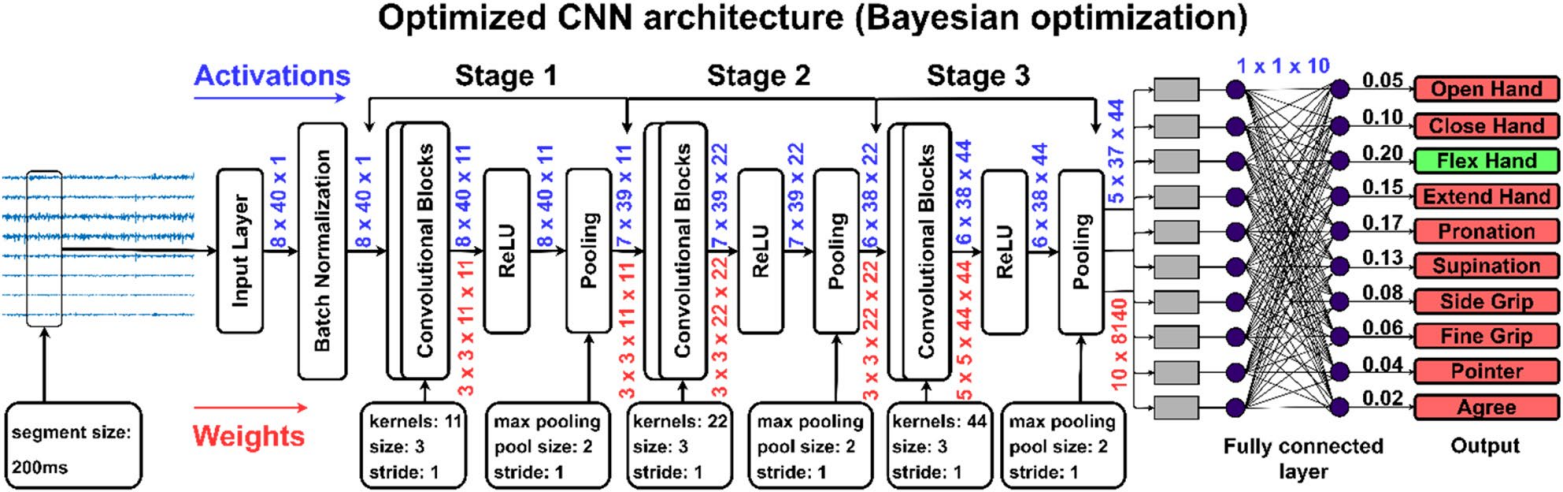
The generalized architecture of CNN. The architecture was designed using the combinations of hyperparameters identified through Bayesian optimization. The number of section depths was set to 2, thus, resulting in the 2 convolutional blocks. Where, each convolutional block is comprised of a convolutional, batch normalization, and ReLU layer.

This study only performed 30 function evaluations (iterations), but it can be increased for better optimisation. However, even if we increase the number of iterations, Bayesian optimisation will automatically cease once it achieves the maximum optimal values. Figure 7 depicts the number of iterations required using Bayesian optimisation to achieve the minimal objective function for all individuals. Although the maximum number of iterations was set to 30, the Bayesian optimisation reached the minimal objective function in less than 20 iterations for most subjects. Furthermore, using Bayesian optimisation, a single set of hyperparameters is evaluated on all participants to investigate the generalizability of the identified hyperparameters. Averaging the learning rate (0.0048), momentum (0.9058), and L2 strength (0.0097) yielded the generalised hyperparameters. In contrast, the mode of the individual section depths has been chosen for the generalised section depth variable, which is 2. Based on these hyperparameters, a single generalised architecture was constructed for all subjects. Figure 8 depicts the designed architecture. The data for each subject were randomly divided into three sections: training (70%), validation (20%), and testing (10%). Image augmentation, as previously described, was used on training sEMG images before model training. An average CER of 0.09 ± 0.03 was obtained on the testing set.

## Discussion

Low generalisation and a high training burden are always major challenges that impede MEC technology adoption. DNNs have the potential to improve generalisation while reducing the MEC system's training burden because of their high generalisation capacity. The developed system should have maximum accuracy while adhering to the real-time MEC restrictions for greater system feasibility and validity in real-time MEC applications. Using CNN as a basic network structure, this paper investigates a gesture recognition framework based on sEMG and Bayesian optimisation for the construction of a MEC system with good generalisation. Furthermore, the effect of segmentation parameters on system performance was explored to determine the appropriate input size for a CNN-based MEC.

The experimental results show that the proposed optimisation method is useful in implementing comprehensive myoelectric control systems. The results revealed a strong association between segmentation parameters and system performance regarding segmentation parameter optimisation. It has been discovered that the overlap segmentation technique considerably outperforms the disjoint segmentation strategy (p-value < 0.05). A distinct trend has also been noted for both types of segmentation approaches. The performance of the CNN degrades as the length of the segment size increases using the disjoint segmentation technique. The segment size of 100 ms demonstrated the best performance, with a mean CER of 25.2%. In contrast, with overlap segmentation, extending the duration of the segment size increases system performance until 200 ms and then begins to decrease. Because overlap segmentation is defined by the length of the segment and the length of the overlap, the influence of overlap size on system performance has also been explored. It has been discovered that increasing the length of overlap size enhances system performance regardless of segment size. The best results were obtained with an overlap size of 80%. Each segmentation strategies exhibit different performance patterns, which could be attributed to the number of input images (sEMG) produced by both segmentation algorithms.

The number of input images reduces as the length of the segment size increases during segmentation. This could be one of the possible explanations for disjoint segmentation. For example, at a segment size of 200 ms, disjoint segmentation produces 208 input images. Overlap segmentation, however, avoids this problem by incorporating samples from the previous section. The number of input images for overlap segmentation is 261 (20% overlap), 348 (20% overlap), 523 (20% overlap), and 1048 (20% overlap). Another key element to consider is capturing enough information from the raw sEMG signal to decipher the intended hand gesture. Because smaller segment sizes create more segments and hence more input sEMG images for training, the optimum performance for overlap segmentation should have been found at the smallest segment size. This is not the case; one of the reasons for the disparity is the information acquired in the segment size to decode the intended hand motion.

According to previous research on this topic, a segment size of at least 200 ms collects enough information to interpret the underlying pattern of the intended hand gesture^7–9^. The findings mentioned above are consistent with prior reports. Furthermore, while this is the first study to investigate DNN-based MEC, the given findings are partially consistent with ML-based MEC systems. Various studies have found that the performance of ML-based MEC systems improves with increasing segment size for both segmentation strategies. The best segment size for disjoint and overlap segmentation has been reported to be 250–300 ms and 275–300 ms, respectively^7–9^. Furthermore, it is reported that for ML-based MEC systems, overlap segmentation with an overlap size of 90% outperforms disjoint segmentation.

**Figure 9:**
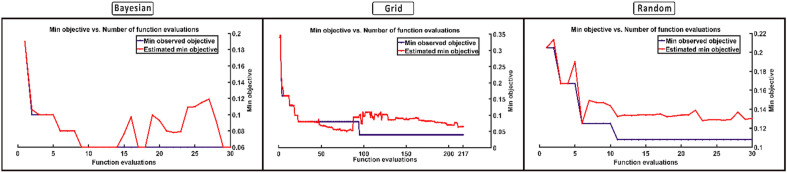
The number of function evaluations required to reach the minimum objective function for Bayesian, random, and grid search optimization methods, correspond to a randomly chosen subject.

The second complementary goal of the research work was to use Bayesian optimisation to optimise the CNN hyperparameters for a MEC system. Because DNN algorithms are data-driven, identifying a suitable set of hyperparameters for any DNN-based MEC system is challenging. As previously stated, various hyperparameter optimisation strategies have been presented. However, finding the ideal set of hyperparameters takes significant time and computer resources. On the other hand, Bayesian optimisation provides a straightforward technique to tackle the challenge of determining the minimum objective function in a relatively short period and with relatively low computational resources. Because the acquisition function aids in swiftly locating the search space, the objective function is only conducted in a specific region space. Since it creates a posterior distribution over the objective function, the Gaussian function is utilised to track analytically. Figure 9 depicts the function evaluations for different optimisation methods, demonstrating how the minimum objective is attained through iterations. The objective function evaluates the samples collected by the acquisition function in each iteration, and the samples are added to the data to update its posterior according to Bayes' theorem. Thus, the hyperparameters are adjusted throughout the layers, and global optimisation is performed across the validation set, lowering the time required and boosting the model's performance.

The results demonstrate the capabilities of Bayesian optimisation in minimising the CER on validation data. The hyperparameters identified on validation data are generalised enough to test the model using the testing dataset to produce comparable results. The average testing CER of 0.08 ± 0.03 on all subjects has been achieved. Table 4 compares different optimisation techniques in terms of the required number of function evaluations, optimisation time, and average CER on the testing set. The comparison with manual, grid and random search methods depicts the superiority of Bayesian optimisation in terms of average CER. The results show that Bayesian optimisation outperforms the random, grid, and manual search optimisation techniques. The grid and random search methods obtained comparable results; however, the required number of function evaluations and optimisation time is significantly large as compared to the Bayesian optimisation.

**Table 4:** The comparison of the proposed (Bayesian) optimization technique with manual, gird, and random search optimization methods.

	Bayesian	Random	Grid	Manual
Function evaluations	30	30	210 ± 13.1	–
Optimization time (Hours)	1.63	2.24	14.03 ± 0.02	–
Testing CER	0.08 ± 0.03	0.12 ± 0.05	0.10 ± 0.03	12.76 ± 4.66

The mentioned number of function evaluations and required optimization time is only for one subject. For manual optimization, the hyperparameters were identified empirically. The last row presents the average CER of all 20 subjects on the testing set.

Finally, we would like to highlight the limitations of the study. Firstly, the study is conducted to optimise an offline MEC system. The previous research shows that the performance of an offline and online MEC differs. Thus, online experimentation should also be conducted to validate the efficacy of the presented optimisation parameters. Secondly, the presented optimisation parameters (segmentation and hyperparameters) have been validated and tested on sEMG data recorded from 20 healthy subjects in a subject-specific configuration. The identified set of optimal segmentation and hyperparameters may induce higher variability in a subject-independent configuration. Thirdly, this study uses the surrogate function based on Gaussian Process to minimise the objective function. In an exploration and exploitation trade-off, the surrogate function drives the proposition of new points to test. An acquisition function that encourages too much exploitation and too little exploration will lead to the model residing only minima it finds first. An acquisition function that encourages the opposite will not stay at a minimum, local or global, in the first place. For better optimisation results, the surrogate function should create a hierarchical process acting as a generative model for domain variables instead of defining a predictive distribution. In the future, Tree Parzen Estimators (TPE) as a surrogate function should be investigated to further enhance Bayesian optimisation's performance to optimise the MEC system's hyperparameters^25^.

## Conclusion

This paper presents a CNN-based, fully optimised MEC framework. sEMG data from 20 healthy volunteers were recorded, corresponding to 10 dynamic and transitional hand gestures. Segmentation parameters (segmentation techniques, segment size, and overlap size) and hyperparameters of the CNN have been optimised. A rigorous empirical experimentation protocol has been implemented to identify the best possible combination of segmentation parameters for the segmentation parameters. Moreover, for optimising the hyperparameters of the CNN, a framework based on Bayesian optimisation has been implemented. The results demonstrate the superiority and generalisation of the identified set of segmentation parameters and hyperparameters compared to the other optimisation techniques. The framework is expected to be further improved and applied in MEC prostheses and exoskeleton devices.

## Data Availability

Data is available on url: https://doi.org/10.34740/KAGGLE/DSV/6845886.
